# Co-delivery of a CD4 T cell helper epitope via covalent liposome attachment with a surface-arrayed B cell target antigen fosters higher affinity antibody responses

**DOI:** 10.1016/j.vaccine.2018.08.014

**Published:** 2018-10-01

**Authors:** Mostafa A. Elbahnasawy, Luke R. Donius, Ellis L. Reinherz, Mikyung Kim

**Affiliations:** a Laboratory of Immunobiology and Department of Medical Oncology, Dana-Farber Cancer Institute, Boston, MA 02115, USA; b Botany and Microbiology Department, Faculty of Science, Al-Azhar University, Nasr City, Cairo 11884, Egypt; c Department of Medicine, Harvard Medical School, Boston, MA 02115, USA; d Department of Dermatology, Harvard Medical School, Boston, MA 02115, USA

**Keywords:** Antibody response, Subunit vaccine, T cell help, Liposome, Vaccine delivery, Helper peptide, LLPC, Long-lived plasma cell, sLACK, liposomes packaged with free LACK peptide, pLACK, liposomes packaged with palmitoylated LACK peptide, iLN, inguinal lymph node, BM, bone marrow, DC, dendritic cell, APC, Antigen-presenting cell, ASC, Antibody-secreting cell, GC, germinal center, Tfh, follicular T helper cell, Tfr, follicular T regulatory cell

## Abstract

Liposomal vaccines incorporating adjuvant and CD4 T cell helper peptides enhance antibody responses against weakly immunogenic B cell epitopes such as found in the membrane proximal external region (MPER) of the HIV-1 gp41 subunit. While the inclusion of exogenous helper peptides in vaccine formulations facilitates stronger and more durable antibody responses, the helper peptide incorporation strategy per se may influence the overall magnitude and quality of B cell target antigen immunogenicity. Both variability in individual peptide encapsulation as well as the potential for liposome surface-associated helper peptides to misdirect the humoral response are potential parameters impacting outcome. In this study, we used MPER/liposome vaccines as a model system to examine how the mode of the potent LACK T helper peptide formulation modulates antibody responses against the MPER antigen. We directly compared liposome surface-arrayed palmitoyl LACK (pLACK) versus soluble LACK (sLACK) encapsulated in the liposomes and free in solution. Independent of LACK formulation methods, dendritic cell activation and LACK presentation were equivalent *in vivo*. The frequency of MPER-specific GC B cells promoted by sLACK was higher than that stimulated by pLACK formulation, a finding associated with a significantly greater frequency of LACK-specific GC B cells induced by pLACK. While there were no significant differences in the quantity of MPER-specific serological responses, the MPER-specific antibody titer trended higher with sLACK formulated vaccines at the lower dose of LACK. However, pLACK generated relatively greater MPER-specific antibody affinities than those induced by sLACK-formulated vaccines. Overall, the results suggest that liposomal surface-associated LACK enhances immunogenicity of LACK through better engagement of LACK-specific B cells. Of note, this is not detrimental to the induction of MPER-specific immune responses; rather, the elicitation of higher affinity anti-MPER antibodies benefits from augmented help delivered via covalent linkage of the pLACK CD4 T cell epitope in conjunction with MPER/liposome presentation.

## 1 Introduction

Modern vaccine strategy is moving away from conventional approaches based on live-attenuated or inactivated forms of microbial pathogens in favor of more safe subunit antigens. This shift focuses the immune response on protective or highly conserved antigenic determinants, that may not elicit a potent response during natural infection or by vaccination with inactivated pathogens. Currently marketed hepatitis B and human papillomavirus vaccines are two successful examples of protein subunit vaccines [1], [2], [3]. Today, effective glycoconjugate vaccines are available for *Haemophilus influenzae* [4], pneumococcus [5], [6], and the meningococcus types A, C, W, and Y [7], [8], [9], [10]. While vaccines have been effective in protecting against pathogens with a low degree of antigen variability, challenges still remain with many of the important pathogens for which no effective vaccine exists including malaria, HIV-1, tuberculosis, and various bacteria [11].

Unlike live-attenuated or inactivated vaccines, which are direct mimics of the natural immunity induced by the pathogens, subunit antigens alone are poorly immunogenic, requiring immunostimulatory molecules to elicit robust humoral and cell-mediated immunity. To elicit long-term humoral immunity, antigenic polysaccharide and peptide vaccines require formulations with MHCII-presented epitopes to engage CD4^+^ helper T cells for induction of robust, high affinity antibody responses [12], [13], [14]. Therefore, protein conjugation or particulate systems along with new technology have been pursued as a means to mediate efficient delivery and activation of innate and adaptive immune cells, shaping the magnitude and quality of the humoral immune response [15], [16]. In that regard, the biophysical properties of vaccine formulation are important determinants of antigen-specific antibody responses affecting the quantity and the quality of immune responses. Immunogenicity of polysaccharide antigens was suppressed by vaccination with multiple glycoconjugate antigens, or by pre-existing immunity against the same carrier due to immunodominance directed to carrier protein [17], [18]. Further, antigen conjugation strategies or encapsulation methods in particulate vaccine formulations have been reported to influence the outcome of immunogenicity of target antigen [19], [20], [21], suggesting the importance of understanding immunological responses influenced by vaccine formulation.

In HIV-1 envelope protein subunit vaccine, immunodominance directed to non-neutralizing epitopes shifts the immune response away from neutralizing antigenic determinants, resulting in suboptimal induction of desirable epitope-specific antibody responses [22]. The membrane proximal external region (MPER) of the HIV-1 gp41 subunit is an attractive vaccine target due to its highly conserved linear sequence targeted by broadly neutralizing antibodies (bNAbs) [23], [24]. MPER is poorly immunogenic during natural infection or by vaccination in the context of envelope protein gp160. While administration of MPER peptides mixed with adjuvant and CD4 T cell helper peptides generated minimal antibody responses, the MPER peptides anchored to the surface of liposome vaccine augmented MPER-specific antibody titers [25], [26]. Although incorporation of CD4 T cell helper peptides are required to induce antigen-specific long-lived plasma cells (LLPCs) and memory B cell responses to peptide-liposome vaccine, the encapsulation efficiency of helper peptides could be suboptimal depending on the physicochemical properties of the peptide sequence and lipid composition [27], [28], [29]. In addition, helper peptides often contain B cell antigen determinants that extraneously compete with target B cell epitopes for help provided by CD4 T follicular helper (Tfh) cells during germinal center (GC) reactions creating a potential epitopic hierarchy. Studies showed that the size and quality of the GC response are directed by Tfh cells which provide growth and differentiation signals to GC B cells and mediate positive selection of high-affinity B cell clones in the GC, thus playing a central role in the production of long-lasting humoral immunity [30], [31], [32], [33].

Utilizing a linear neutralizing epitope targeted MPER peptide/liposome vaccine as a model system, we aimed to define the B cell response competition and interplay following immunization with MPER/liposomes packaged with sLACK for encapsulation and pLACK for covalent linkage of LACK to the liposome surface. The LACK_156-173_ peptide, a well characterized immunodominant CD4 T cell epitope presented by the I-A^d^ (MHC class II) molecule, was derived from the *Leishmania major* RACK-like homolog of the WD protein family [34]. While the magnitude of MPER-specific serological antibody responses is independent of LACK formulation per se, higher affinity antibody induction facilitated by pLACK compared to sLACK suggests that the elicitation of high affinity protective antibody may benefit from co-delivery of lipid-anchored helper peptides with B cell antigen derived from pathogens with a high mutation rate.

## 2 Materials and methods

### 2.1 Animal care and use

All animal procedures were performed according to protocols approved by the Dana-Farber Cancer Institute and Harvard Medical School Animal Care and Use Committee Institutional Review Board. 8–10 week old naïve, wild type, female BALB/c mice were purchased from Taconic Biosciences (Hudson, NY, BALB/cAnNTac) and maintained in a specific pathogen-free facility at Dana-Farber Cancer Institute.

The following primary mouse samples were obtained: blood via tail vein puncture, inguinal lymph nodes (iLNs), spleens, and bone marrow (BM). Single-cell suspensions of the combined iLNs were generated by mashing lymph nodes through a 70 µm strainer into FACS buffer (0.5% BSA 2 mM EDTA PBS). Splenocytes were similarly mashed through a strainer; however, followed by a red blood cell lysis step before being resuspended in FACS buffer. BM was collected from the combined femurs and tibias by removing the ends of the bones and flushing the cells out with PBS. BM red blood cells were further lysed and the cells were resuspended in FACS buffer. Sera was collected from tail vein by isolation of ∼50 μl blood from gently-warmed (under a heat lamp) mice. Blood was maintained at room temperature and was allowed to coagulate. Serum was then isolated by centrifugation for 5 min in a microcentrifuge at high speed. Supernatant was collected and stored at −20 °C until assayed.

### 2.2 Liposomes and peptides

MPER/liposomes were prepared as described previously [35]. In brief, the following components were mixed: MPER peptide, monophosphoryl lipid A (MPLA), 1,2-dioleoyl-sn-glycero-3-phosphocholine (DOPC), 1,2-dioleoyl-sn-glycero-3-phospho-(1′-rac-glycerol) (DOPG) and 1,2-dimyristoyl-sn-glycero-3-phosphocholine (DMPC) (Avanti Polar Lipids, Alabaster, AL) with or without N-terminally palmitoylated-LACK (pLACK) for the pLACK formulated MPER/liposome preparation. For free LACK (sLACK) formulated MPER/liposomes, organic solvents were fully evaporated and the following day the liposomes were rehydrated in PBS with the addition of sLACK. In addition to the sLACK and pLACK formulations above some liposomes were formulated with sLACK added following extrusion (post-extrusion) to ensure no encapsulation. For ELISA and calcium flux assays, liposomes consisted of 1:50 or 1:1000 palmitoylated peptide in DOPC:DOPG (4:1) lipids with 0.2% biotinylated polyethylene glycol (PEG) 2000. ELISPOT liposomes were formulated identically with exclusion of the PEG biotin. For fluorescent liposomes a peptide:lipid ratio of 1:200 was used with 4:1 DOPC:DOPG and either 1% biotin-polyethylene glycol-DSPE or 1% carboxyfluorescein-DOPE (all lipid reagents from Avanti Polar Lipids; Alabaster, AL) along with 3% or 4% polyethylene glycol (2000)-DOPE, respectively.

As described by others the LACK (LACK_156–173_) sequence was (ICFSPSLEHPIVVSGSWD) [36]. The MPER peptide was an N-terminally palmitoylated MPER_662-683_ peptide (ELDKWASLWNWFNITNWLWYIK) synthesized at the Massachusetts Institute of Technology Biopolymers and Proteomics Core Facility (Boston, MA). For immunization studies, mice (5 mice per group) were administered with pLACK or sLACK formulated MPER/liposome vaccine (50 µl/injection, 2.52 mg of total immunization liposomes per mouse) intradermally at day 0 and again at day 30. MPER/liposomes for immunization were formulated as above and injected into mice to deliver palm-MPER at 1:200 with lipid, 17.5 μg of MPLA, and 40 μg of LACK if not noted otherwise.

### 2.3 *In vitro* 4E10-WEHI cells

4E10-expressing WEHI231 B cells were generously provided by the Nemazee lab [37] and cultured in advanced DMEM medium supplemented with 1X glutamax, penicillin-streptomycin, β-mercaptoethanol (all from Life Technologies), and 5% FBS (Sigma-Aldrich, St. Louis, MO). Surface 4E10 expression was induced by doxycycline (1 µg/ml) treatment overnight at 37 °C. The following day cells were washed and utilized for *in vitro* experiments. The expression of 4E10 BCR was verified by detection of the 4E10 human kappa light-chain (hC_k_) with anti-hC_k_.

Supplementary data associated with this article can be found, in the online version, at https://doi.org/10.1016/j.vaccine.2018.08.014.

To quantify presentation efficiency of LACK, 4E10-WEHI or uninduced cells were resuspended at 0.5 million cells/mL and treated with various MPER/liposomes (100 µg/mL), formulated as for the pLACK immunization liposomes, for 60 min. After an additional incubation for 6 h, the cells were then harvested, stained with a biotinylated LACK:I-A^d^-specific monoclonal antibody (clone 2C44; produced and purified from hybridoma cells that were generously provided by the Mougneau lab [36]) and followed by fluorescent streptavidin and antibody incubation (see Supplement Table S1) and flow cytometric analysis.

**Supplementary Tables S1–S4:** 

MPER-specific BCR stimulatory strength was assessed by Ca^2+^-flux of 4E10-WEHI cells. Cells were washed with serum/phenol-free RPMI and resuspended at 1 × 10^7^ in RPMI with 3 µg/ml Fluo-4AM calcium sensitive dye. Cells were protected from light and incubated at 37 °C for 1 h with inversion mixing at 30 min.  > 5X excess cold RPMI was used to wash cells. Additionally, cells were washed 2X with 0.1% BSA HBSS and resuspended in 0.1% BSA HBSS (Calcium chloride supplemented) at 1 × 10^6^ cells/ml. 1 ml aliquots were warmed to 37 °C in a water bath for 10 min prior to analysis. Flow cytometric analysis was performed with a 30 s baseline then spiking in MPER liposomes (50 μg), anti-IgM (10 μg), or PBS and analyzing 230 s more. Expression was always confirmed by surface expression of the 4E10 anti-human kappa chain.

### 2.4 Flow cytometry

Single cell suspensions were prepared from draining iLNs, where described, by mashing through 70 μm nylon mesh cell strainers (Corning, Durham, NC). Cells were resuspended in PBS with FACS buffer. In general, 5 million cells per sample were distributed in wells of a conical-bottom 96-well plate and sequentially stained for 20 min on ice with antibodies in 100 µl volumes of antibody mix as described in supplemental tables. All cells were stained for 30 min in PBS with Fixable Viability EF506 (Life Technologies). All antibody staining was carried out by resuspending cells in 50 µl Fc block and then mixing in 50 µl of detection antibodies. In exception, cells were incubated with anti-CXCR5 and anti-PD1 antibodies for 1 h on ice. Similarly, staining for MPER- and LACK-specific B cells was done by incubating cells with 100 µg/ml fluorescent MPER and LACK (or corresponding bare) liposomes for 1 h on ice (see Supplement Tables S2 and S3). Between each incubation cells were washed 2X 100 µl of FACS buffer. All analyses, including for 4E10-WEHI experiments were performed on a BD LSR Fortessa flow cytometer and analyzed using FlowJo software (10.4.1) (Tree Star).

In experiments where dendritic cells were assessed the iLNs (draining) were harvested and dissociated in collagenase/DNase (10 mg/ml and 5U/ml, respectively) containing 10% FBS RPMI. Lymph nodes were maintained at 37 °C and 5% CO_2_ with fine dissociation and additional media added every 15 min for one hour. Cells were then stained with an antibody cocktail (see Supplement Table S4).

### 2.5 ELISA quantification of antigen-specific antibody response

MPER- and LACK-specific ELISAs were performed as before [35]. In brief, 96-Well Immulon 2HB Plates (Thermo Fisher Scientific) were coated overnight with streptavidin in PBS (50 µl/well) at 4 °C. Wells were washed, blocked, and coated with peptide/liposomes (palmitoylated MPER or LACK). Wells were washed again and incubated overnight at 4 °C with serially diluted sera. The following day, serum was removed, the plate was washed, incubated with HRP-conjugated secondary antibody, re-washed, the signal was developed with *o*-phenylenediamine (OPD) solution (see Supplement Table S5), and the absorbance was measured at 490 nm.

### 2.6 ELISPOT quantification of MPER-specific ASCs

Quantification of MPER-specific antibody-secreting cells (ASCs) was performed using ELISPOT assay, as previously described [35]. Briefly, 96-well high protein-binding Immobilon-P (PVDF) membrane plates (EMD Millipore, Billerica, MA) were activated with 35% ethanol, washed, and coated with 100 µl per well of 100 µg/ml peptide/liposomes and incubated overnight at 4 °C. Plates were washed, blocked with 200 µl/well 1% BSA-PBS for at least 4 h, followed by washing and blocking with growth media (10% FBS, 1X glutamax, 1X penicillin/streptomycin, 1x 2-mercaptoethanol RPMI) for at least an hour. Cells were quantified, resuspended to 1 × 10^7^ cells/ml, and 50 µl volumes (500,000 cells) were added to wells in triplicate or quadruplicate. Hybridoma cells (M1) which secrete a C-terminal MPER-specific antibody and bNAb 2F5-expressing cells were always plated as controls. Plates incubated overnight at 37 °C and 5% CO_2_ in a humidified chamber. The following day, plates were washed and spots were visualized by alkaline phosphatase secondary antibody staining.

### 2.7 Statistical analysis

All graphing and statistical analyses were performed using GraphPad Prism V7.02 (GraphPad Software).

## 3 Results

### 3.1 MPER-specific engagement of the BCR mediates MPER/liposome vaccine uptake and LACK/MHCII (I-A^d^) presentation

**Fig. 1:**
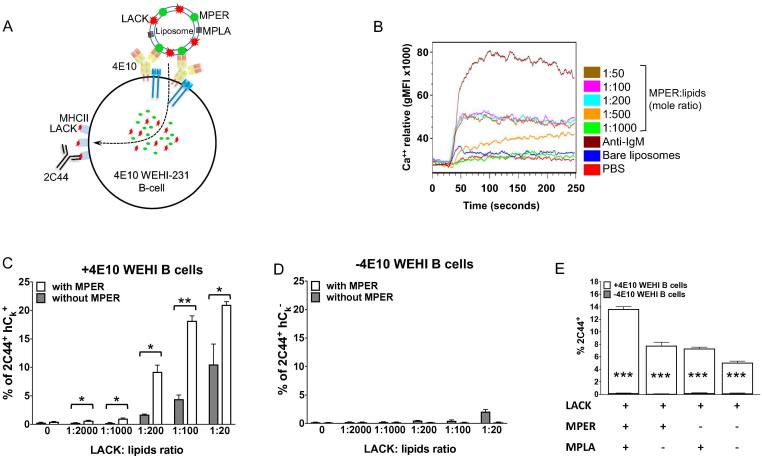
MPER-specific discrimination and engagement by the BCR mediates co-delivery of LACK for MHCII presentation. (A) Schematic of pLACK formulated MPER/liposome engagement by BCR of 4E10 BNAb-expressing WEHI-231B cells and the subsequent detection of MHCII(I-A^d^)/LACK complex by antibody 2C44. (B) Ca^2+^ mobilization analysis of 4E10 bNAb-expressing WEHI-231B cells upon stimulation with the various MPER/liposomes. MPER peptides arrayed on the surface of liposome at different molar ratios of peptide to lipid (1:50–1:1000) were tested. Colored lines represent Fluo4-AM fluorescence in the presence of Ca^2+^ ions following stimulation with different MPER ratios and for positive anti-IgM F(ab)_2_ (dark brown) and for negative bare liposome (blue) and PBS (red) control treatments. (C and D) Quantification of LACK:I-A^d^ complexes on the surface of WEHI-231B cells expressing 4E10 BCR (human kappa chain^+^; hC_k_^+^) (C) or lacking 4E10 BCR (hC_k_^−^) (D) as determined by flow cytometry using antibody 2C44. MPER/liposomes (white bars) or bare liposomes (gray bars) formulated with pLACK at different molar ratios of LACK peptide to lipid were incubated with WEHI cells for 1 h, followed by a 6 h incubation for processing/presentation, and detection with 2C44. (E) Synergistic effect of MPLA on the I-A^d^/LACK presentation by 4E10 expressing WEHI cells. pLACK/liposomes containing MPER or MPLA or MPER and MPLA as denoted by (+) signs in table were incubated with 4E10-expressing cells (white bars) and 4E10-negative cells (gray bar in-lays) for 1 h with various liposomes packaged with 1:100 pLACK to lipid followed by a period of time for processing as described in *Materials and Methods*. Results are representative of three independent experiments. Bars represent the mean with plus/minus SEM shown. (*) p < 0.05, (**) p < 0.01, or (***) p < 0.001 by unpaired student’s T-test. (For interpretation of the references to colour in this figure legend, the reader is referred to the web version of this article.)

We first determined the maximal density of MPER and pLACK on the surface of liposomes in an *in vitro* assay utilizing a mouse B cell lymphoma cell line expressing MPER-specific bNAb 4E10 (4E10 WEHI) (Fig. 1A) [37]. Since clustering of B cell antigen receptors (BCRs) through engagement of multivalent antigens induces much stronger downstream activation and survival signals to the B cells, promoting strong antibody responses, the optimal density of MPER peptides was determined by measuring the strength of intracellular calcium (Ca^2+^) flux as a readout following stimulation with MPER/liposomes formulated with a range of peptide to lipid ratios. A previous study identified a significant shift in the *in vivo* humoral immune response at a 1:1000 molecular threshold [26]. Utilizing the fine resolution of Ca^2+^ flux we tested 4E10 BCR engagement at a range MPER to lipid from 1:50 to 1:1000. A maximum threshold for MPER/liposome stimulation was reached at a 1:200 (Fig. 1B). Activation was strictly specific for MPER, as bare liposomes and PBS initiated very minimal Ca^2+^ flux. Given an equivalent Ca^2+^ flux exhibited at the MPER:lipid ratio higher than 1:200, the density of MPER on the surface of liposomes was fixed at the 1:200 ratio in the following studies. Next, we determined BCR-mediated uptake of pLACK formulated MPER/liposome and the subsequent LACK presentation by 4E10 WEHI cells by quantifying the LACK:I-A^d^ complex-specific antibody 2C44 binding (Fig. 1A) [36]. LACK:I-A^d^ presentation was strongly MPER- and LACK-dose-dependent (Fig. 1C) and BCR (4E10)-dependent (Fig. 1D). Note that MPER-independent LACK presentation is still present on 4E10 WEHI cells due to 4E10 binding to lipids as is well characterized previously [38]. Since MPER/liposome vaccine formulation includes the cell surface expressed TLR4 agonist MPLA, we also tested the effect of surface receptor mediated LACK presentation by 4E10 WEHI cells. The LACK presentation was higher (∼13% 2C44^+^) with MPLA containing MPER/liposome than MPER/liposomes without MPLA (∼7% 2C44^+^) (Fig. 1E), indicating a synergistic effect of MPLA on BCR-dependent LACK presentation. Given the endocytic potential of TLR4, the complete absence of LACK presentation without 4E10 expression may be explained by the lack of CD14 expression on the surface of WEHI231 cells [39]. TLR4 mediated-endocytosis requires co-operation with CD14 [40].

### 3.2 Both pLACK and sLACK formulated liposome vaccines efficiently delivered LACK peptides to APCs *in vivo*

**Fig. 2:**
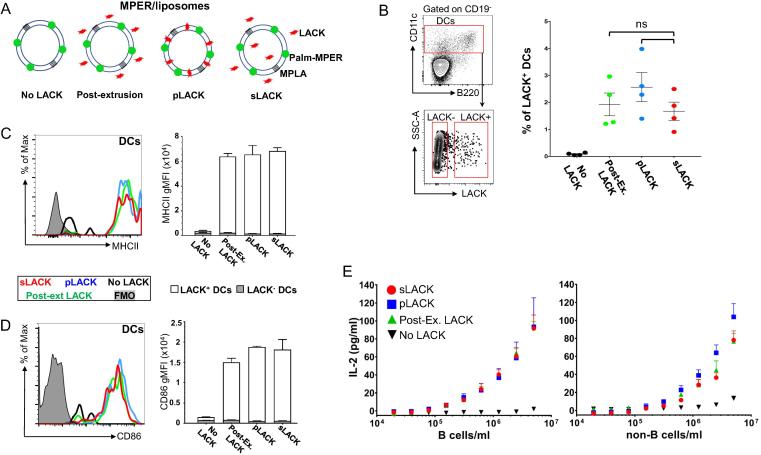
Both pLACK and sLACK formulated MPER/liposome vaccines efficiently deliver LACK peptides to antigen presenting cells *in vivo*. (A) Schematic representation of various incorporation methods for LACK into MPER/liposome: (1) “post-extrusion”, sLACK added in extruded MPER/liposome solution, (2) “pLACK”, surface associated palmitoylated LACK to MPER/liposome, (3) “sLACK”, free and encapsulated sLACK in MPER/liposome solution utilized to test the delivery efficiency to APCs following immunization. (B) *In vivo* quantification of LACK:I-A^d^ complexes on the DCs by 2C44 monoclonal antibody 1 day post-immunization. Gating strategy (left) and the frequency of LACK^+^ DCs (CD19^−^ CD11c^+^) in draining iLN was determined by flow cytometry analysis using 2C44 (right) to compare the efficacy of different LACK formulation methods (1–3 above; green, blue and red, respectively) on the LACK delivery to DCs. (C and D) Upregulation of MHCII (C) and CD86 (D) was quantified from the geometric mean fluorescent intensity (gMFI) of surface expression (representative histograms in left panels) for LACK^+^ DCs and LACK^−^ DCs as determined in (B) and graphed in the right panels (open bars and filled inlay, respectively). (E) IL-2 secretion by LACK-specific T cell hybridomas upon incubation with B cells (left) or the DC containing non-B cell fraction (right) in post-immunization *ex vivo* cultures. Purified B cells and the remaining non-B cells from draining iLN of 1 day immunized mice were cultured with LACK-specific T cell hybridoma cells overnight, and supernatant IL-2 was quantified by ELISA. Points represent mean with error bars denoting SEM. Results are representative of two independent experiments, each with at least four mice per group. (ns) = not significant by the unpaired student’s *t*-test. (For interpretation of the references to colour in this figure legend, the reader is referred to the web version of this article.)

Next, we designed three different LACK incorporation methods: (1) soluble LACK mixed with MPER/liposome after liposome extrusion (post-extrusion) (2) surface associated palmitoylated-LACK in MPER/liposome (pLACK) (3) mixture of liposome encapsulated and free soluble LACK in the MPER/liposome solution (sLACK) (Fig. 2A). The 40 μg dose of LACK in each formulation was selected based on results above for the maximal loading density on the surface of MPER/liposome vaccine as the 80 μg dose of pLACK resulted in liposome aggregation (data not shown). The effects of the three different LACK formulations on the activation of dendritic cells (DCs) and their LACK presentation were then assessed at 24 h post-immunization of BALB/c mice. LACK:I-A^d+^ CD11c^+^ DCs in the draining iLN constituted ∼2% of the DC population (2.5, 1.6, and 1.9% for pLACK, sLACK, and post-extrusion, respectively), while mice immunized without LACK resulted in a low background threshold of only 0.08% DC staining by 2C44 by flow cytometry (Fig. 2B). The upregulation of the MHCII and CD86 on DCs was equivalent between all three different groups as well (MHCII gMFI ∼ 6000 and CD86 gMFI ∼ 1700; Fig. 2C and D; representative histograms in left panels and quantification in right panels). However, LACK presentation by DCs was correlated with significantly higher MHCII and CD86 expression compared to that in LACK negative DC populations (Fig. 2C and D, open bars versus inlaid filled bars). To independently estimate the levels of LACK:I-A^d^ complexes at the cell surface, fractionated B cells or non-B cells from iLN cells of 1 day immunized BALB/c mice were incubated with LACK-specific LMR7.5 T cell hybridoma cells. Both the B cell and non-B cell fractions stimulated LMR 7.5 hybridomas to secrete equivalent amounts of IL-2 independent of LACK liposome formulation (Fig. 2E). Overall these analyses suggest that the form of LACK delivery to APCs does not affect the efficacy of LACK presentation by APCs at 24 h post-immunization.

### 3.3 LACK-specific GC B cells are significantly enhanced by covalent pLACK association with the liposome surface

**Fig. 3:**
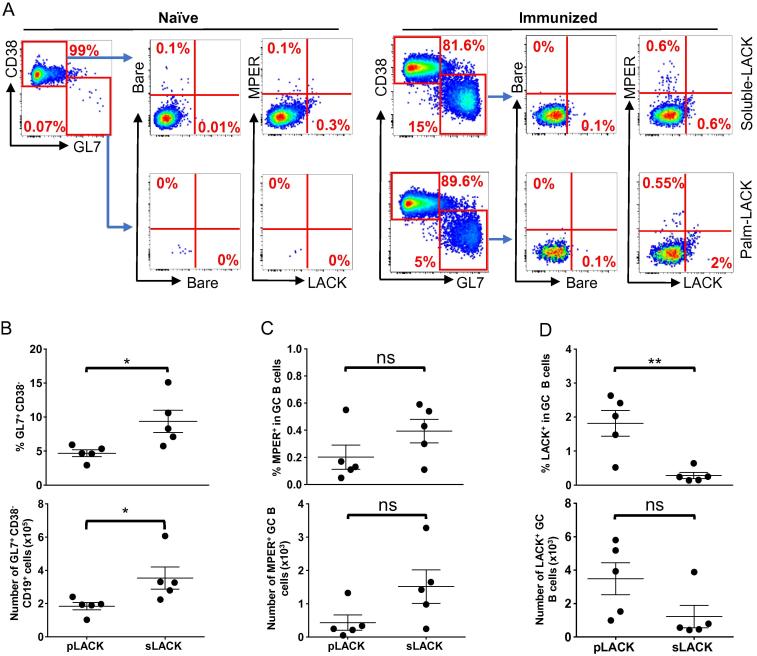
pLACK formulated MPER/liposome vaccine induces significantly higher frequency of LACK- specific GC B cells compared to that of MPER- specific GC B cells. (A) Gating strategy for MPER- and LACK-specific GC B cells (CD19^+^ CD38^−^ GL7^+^) by flow cytometry on day 14 after immunization. Palmitoylated-MPER/liposome labeled with BV421 (1:200 peptide to lipid ratio) and palmitoylated-LACK/liposome (1:200) labeled with FITC were used to determine the frequency of MPER- and LACK-specific GC B cells. GC B cells binding bare liposomes were used in conjunction with the antigen-arrayed liposomes for antigen-specific quantification. Naïve mouse derived cells were used to guide gating decisions. (B) Total GC B cell percentage (top) and the total number of cells was quantified (bottom). (C and D) Frequencies of MPER- specific (C, top) and LACK-specific (D, top) GC B cells. The total number of MPER- (C, bottom) and LACK-specific cells (D, bottom) were determined after subtraction of cell numbers binding to bare/liposomes. Brackets represent the means of five mice per group from one of two independent experiments. Bars represent SEM. (*) p < 0.05 and (**) p < 0.01 by the unpaired Students *t*-test. Results are representative of two independent experiments. All mice were administered intradermally with sLACK or pLACK formulated MPER/liposome and draining iLNs were taken out 14 days post-immunization for analysis.

**Fig. 4:**
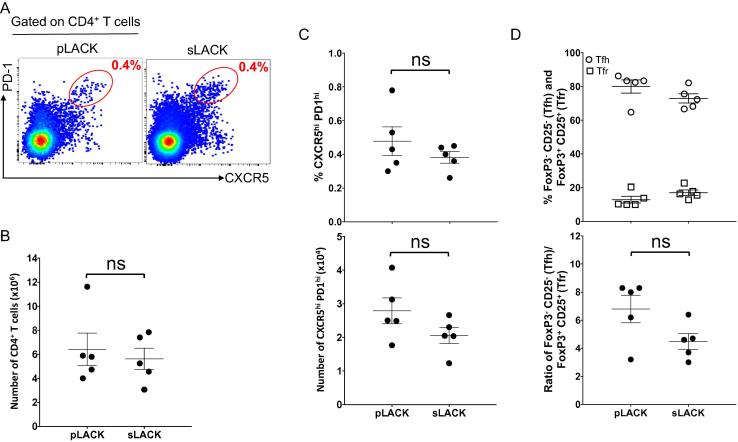
sLACK- and pLACK-loaded MPER/liposomes promote equivalent Tfh responses. (A) Flow cytometric analysis of the CD4^+^ PD-1^hi^ CXCR5^hi^ Tfh cells from the draining iLNs at 14 days post-immunizations same as in Fig. 3. (B) The total number of CD4^+^ T cells. (C) The percent of PD-1^hi^ CXCR5^hi^ cells, as shown in A, was quantified (top) and the total number of these cells was calculated (bottom). (D) The percent of Tfh cells with a classical stimulatory activity (FoxP3^−^ CD25^−^; circles) compared to those with a regulatory, Tfr, activity (FoxP3^+^ CD25^+^; squares) was determined from the subset of PD-1^hi^ CXCR5^hi^ (top). The total number of Tfh and Tfr cells was determined and the ratio is shown (bottom). Results are representative of two independent experiments with five mice per group done in parallel from the same mice as in Fig. 3. The lines represent the means with error bars for SEM shown. ns = not significant by the unpaired student’s *t*-test.

In a previous study, MPER-specific IgG titers following MPER/liposome immunization were reduced by >90% in CD4^+^ T cell-depleted animals, indicating the essential role of CD4 T cell help for the induction of robust MPER-specific antibody responses [26]. Given the high incorporation efficiency of liposome surface exposed pLACK via covalent linkage compared to that of surface absorbed LACK by post-extrusion mixing method, >90% vs 35%, respectively by mass spectrometry analysis, (data not shown), the surface exposed pLACK formulation method was further selected to compare the effect of liposome encapsulation of sLACK on the MPER-specific cellular and antibody responses. GC B cells and Tfh cells typically reflect the magnitude and quality of the antigen-specific B cell response. To further investigate the influence of LACK formulation on the GC response, the mice were immunized with MPER/liposomes formulated to deliver 40 μg/mouse of either pLACK or sLACK and then draining iLNs were isolated 14 days later to quantify total GC B cells (CD19^+^GL7^+^CD38^−^) and MPER-specific GC B cells (Fig. 3A) by flow cytometry. The percent and total number of GC B cells (Fig. 3B; top and bottom panels, respectively) is greater following immunization with sLACK formulated MPER/liposomes than that with pLACK. Next, we measured the frequency of MPER- and LACK-specific GC B cells using palmitoylated-MPER/liposome labeled with BV421 and palmitoylated-LACK/liposome labeled with FITC (Fig. 3A). The “bare” liposomes were utilized as a negative control to subtract background from antigen-specific staining. The detection specificity for MPER- and LACK-specific GC B cells was further verified by the expansion of antigen-specific B cells in iLNs of representative vaccine immunized mouse (Fig. 3A, bottom) compared to that of naïve mouse B cells (Fig. 3A, top). Surprisingly, the frequency and total MPER^+^ GC B cell population was not significantly affected by the LACK formulation (Fig. 3C); although, a trend suggests a relatively higher frequency of MPER-specific B cells induced by sLACK (0.4%) compared to that by pLACK (0.26%) formulation (Fig. 3C, top), which corresponded to 1515 and 433 MPER+ GC B cells, respectively (Fig. 3C, bottom). However, the pLACK formulation generated a significantly higher frequency of LACK-specific GC B cells (1.8%) compared to that by sLACK formulation (0.28%), likely influencing MPER-specific GC B cells generation (Fig. 3D, top). Likewise, the number of LACK-specific GC B cells generated by pLACK was higher than that generated by sLACK (Fig. 3D, bottom), although no significant difference was observed statistically. Next, the Tfh cells (CD4^+^PD-1^hi^CXCR5^hi^) (Fig. 4A) that guide GC B cell maturation were quantified to assess the quality of sustained LACK presentation with sLACK or pLACK formulations. No significant difference in total CD4 T cell number (Fig. 4B), Tfh frequency (Fig. 4C, top), or total Tfh number (Fig. 4C, bottom) was present between the two formulations. Further, no statistically significant difference in the balance between the stimulatory (Tfh; FoxP3^−^CD25^−^) and suppressive (Tfr; FoxP3^+^CD25^+^) subsets of the PD-1^hi^CXCR5^hi^ population was formed (Fig. 4D) as evidenced by the frequency of the populations (Fig. 4D, top) or the ratio of the total number of FoxP3^−^CD25^−^ to FoxP3^+^CD25^+^ (Fig. 4D, bottom). Nevertheless, the greater number of Tfh cells and the reduced Tfr cells generated by pLACK formulation compared to those by sLACK trended along with a corresponding increase in the ratio of stimulatory Tfh cells to inhibitory Tfr cells in the GCs of pLACK immunized mice.

### 3.4 Quality of the MPER-specific antibody response was influenced by LACK formulation

**Fig. 5:**
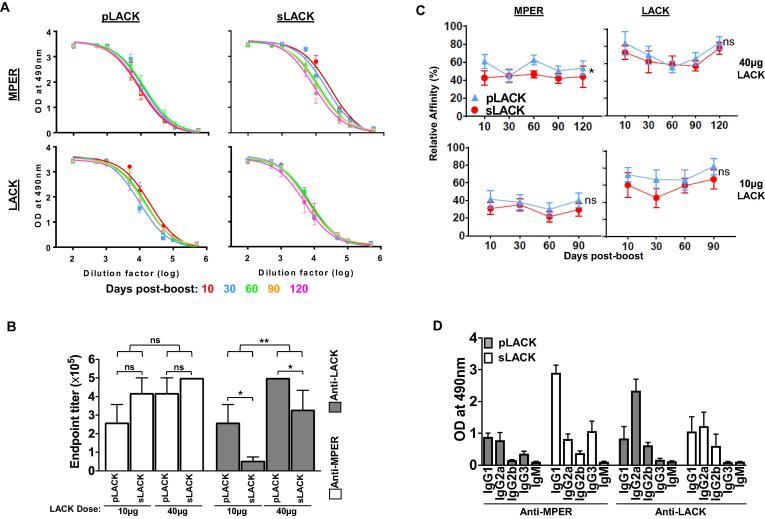
sLACK- and pLACK-formulated MPER/liposome vaccines elicit qualitatively different MPER-specific antibody responses. (A) Anti-MPER IgG (top) and anti-LACK IgG ELISA titers (bottom) elicited by pLACK formulated MPER/liposome vaccine (left) were determined at various time points following a boost injection and compared with those elicited by sLACK formulated MPER/liposome vaccine (right). (B) Dose effect of LACK on the anti-MPER and the anti-LACK IgG ELISA titers at 30 days post-boost. The effect of the dose (black lines) and formulation (comparison of bracketed groups) was tested within each antigen specificity by 2-way ANOVA with (*) = p < 0.05, (**) = p < 0.01, and (ns) = not significant. (C) The kinetic trend in relative affinities for MPER- and LACK-binding IgG at various time points following boost was assessed by ELISA for pLACK- (blue) and sLACK-formulated (red) MPER/liposomes. Absorbance values used for calculating the ratio were specifically determined by finding the absorbance at the high density MPER or LACK/liposome (peptide to lipid ratio of 1:50) EC50 and using that EC50 dilution to find the absorbance given on the low density MPER or LACK/liposome (1:1000) curve. The relative affinity was determined for both the 40 µg LACK/mouse (top left and right) as in (A) as well as a reduced 10 μg LACK/mouse (bottom left and right). As in (B), the temporal trends and effects of sLACK and pLACK formulation were assessed by 2-way ANOVA. Statistics for sLACK versus pLACK are shown (*p < 0.05 or ns = not significant) (D) Analysis of anti-MPER and anti-LACK immunoglobulin subclasses in the sera of mice immunized with sLACK (white bars) or pLACK (gray bars) formulated MPER/liposome vaccine (30 days post-boost; 40 µg LACK/mouse). Bars represent mean absorbance of antigen-specific immunoglobulin isotype at 1:10000 sera dilution. All points in panels of A, B, C and D represent means of five mice per group, unless individuals are shown, with error bars representing SEM. All graphs represent mice immunized with sLACK or pLACK formulated MPER/liposome vaccine intradermally two times at a 30 day interval with a 10 µg or 40 µg dose of LACK/mouse as indicated. (For interpretation of the references to colour in this figure legend, the reader is referred to the web version of this article.)

Given the generation of LACK-specific GC B cells, we assessed the extent to which pLACK and sLACK may affect the induction of the MPER-specific antibody response. While after two immunizations, both pLACK (40 μg dose) and sLACK (40 μg dose) formulated vaccines generated durable, strong anti-MPER (Fig. 5A, top left and right) and anti-LACK antibody responses (Fig. 5A, bottom left and right), kinetic analysis showed modest reduction of anti-MPER antibody titer over time elicited by sLACK compared to that induced by pLACK formulation (Fig. 5A, top right). Nevertheless, overall MPER-specific antibody titer remained slightly higher in immune sera generated by sLACK than that by pLACK (endpoint titer 4.5 × 10^5^ vs 3.5 × 10^5^) at 120 days after boost (Fig. S1A, top). An opposite trend was shown with anti-LACK antibody titers (5 × 10^5^ by pLACK vs 2.5  × 10^5^ by sLACK) (Fig. S1A, bottom). However, the pLACK formulation induced significantly higher anti-LACK antibody titer (4.8 fold) compared to that by sLACK formulation at 10 μg dose of LACK. At the same time, the elicitation of the anti-MPER antibody response was modestly reduced by pLACK formulation relative to that induced by sLACK (Fig. 5B), indicating the anti-MPER antibody response is modulated by LACK dose and formulation. To compare the relative affinities of antibodies for MPER and LACK between sLACK and pLACK immunized groups, we utilized the difference in binding of antibodies to the peptides at the low density (1:1000 peptide:lipid ratio) versus at the high density (1:50 ratio) as we had previously established for determining relative affinity [35]. Over time, the antibody affinity for the MPER and the LACK from pLACK-immunized groups always trended higher than that from sLACK-immunized groups, regardless of LACK dose (Fig. 5C). In addition, the generation of relatively higher affinity antibody for LACK compared to that for MPER was independent of LACK dose. On the other hand, overall affinity maturation of MPER-specific antibodies was positively correlated with LACK dose as shown in Fig. S1B.

**Supplementary Fig. 1:** Comparison of temporal and dose kinetics within sLACK and pLACK immunized mice. (A) Anti-MPER IgG (top) and anti-LACK (bottom) IgG ELISA titers quantified as the reciprocal of the dilution generating signal two times above background, elicited by 40μg sLACK-formulated (red circles) MPER/liposome vaccine compared with those elicited by 40μg pLACK-formulated (blue triangles) MPER/liposome vaccine at 10, 30, 60, 90, and 120 days post-boost. (Data is derived from curves shown in Fig. 5A) (B) Direct comparison of the effect of LACK dose (10μg, dashed line and 40μg, solid line) on MPER-specific IgG affinity maturation. Data is identical to that shown in (Fig. 5C); however, plotted data and statistics rearranged for direct comparison of dose rather than LACK formulation. Statistical analysis by two-way ANOVA with p-value for dose comparison shown (**p<0.01). Top graph shows pLACK (blue triangles) and bottom is sLACK (red circles) results. Absorbance values used for calculating the ratio were specifically determined by finding the absorbance at the high density MPER or LACK/liposome (peptide to lipid ratio of 1:50) EC50 and using that EC50 dilution to find the absorbance given on the low density MPER or LACK/liposome (1:1000) curve.

Next, to determine the qualitative influence of the different LACK formulation methods on B cell class-switch, isotype-specific ELISAs were performed on MPER- and LACK-specific immune sera. While sLACK induced MPER-specific IgG subclass responses skewed towards IgG1 isotype, balanced IgG1 and IgG2_a_ isotype antibody responses specific to LACK were elicited. On the other hand, the pLACK formulated vaccine produced equivalent IgG1 and IgG2_a_ isotype antibodies specific to MPER but a dominant IgG2_a_ antibody response specific to LACK (Fig. 5D).

### 3.5 Both sLACK and pLACK formulations generated equivalent MPER-specific antibody secreting LLPCs in BM

**Fig. 6:**
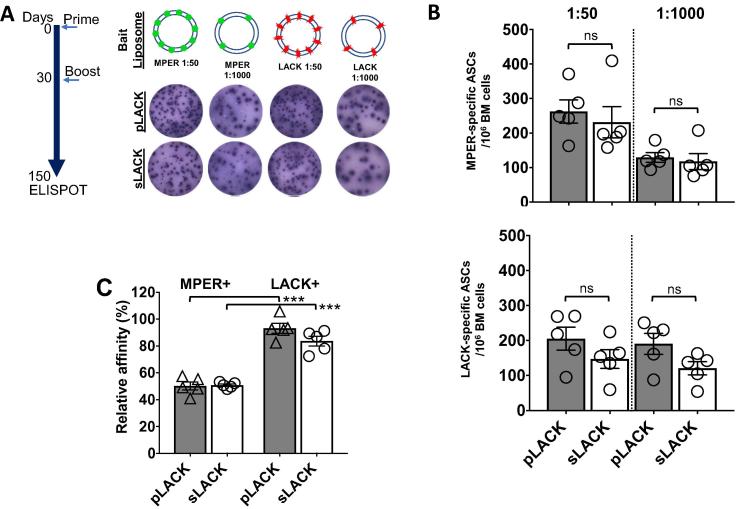
Generation of long-lived MPER- and LACK-specific plasma cells in bone marrow promoted by sLACK and pLACK formulated MPER/liposome vaccine (A) Experimental timeline to define long-term bone marrow MPER- and LACK-specific ASCs. ELISPOT analysis was performed at 150dpi and examples of high density (1:50 ratio, peptide:lipid) and low density (1:1000) MPER or LACK/liposome reactive spots from pLACK- and sLACK-formulated liposomes as shown. (B) Quantification of MPER- (top) and LACK-specific ASCs (bottom) per million bone marrow cells (1:50, left columns; 1:1000, right columns) from sLACK (open bars) and pLACK (filled bars) formulated MPER/liposome. (C) Relative affinity of MPER- and LACK-specific bone marrow ASCs. ns = not significant and ***p < 0.001 by the unpaired Students *t*-test. Mean ±SEM shown for n = 5 mice (individual circles).

To quantify the number of the MPER- and LACK-specific antibody secreting cells (ASCs) persisting long-term, ASCs in BM at 150 days post-immunization were estimated by ELISPOT assay against MPER/liposome- or LACK/liposome-coated on the plates (Fig. 6A). The frequency of the MPER-specific ASCs between the two different groups of mice was equivalent to ∼225–250 ASCs/10^6^ cells at the peptide to lipid ratio of 1:50 (Fig. 6B; top) and ∼100 ASCs/10^6^ cells at 1:1000. Further, mice immunized with the pLACK formulation generated ∼200 ASCs/10^6^ of LACK-specific ASCs compared to ∼150 ASCs/10^6^ in mice immunized with sLACK (Fig. 6B; bottom). The relative affinity calculated from these demonstrated that the extent of affinity maturation of MPER- and LACK-specific LLPCs was achieved equivalently between groups immunized with sLACK and pLACK formulated vaccines (Fig. 6C). However, a considerable advantage in affinity maturation against LACK (∼90%) than MPER (∼50%) was evident, suggesting innate affinity of BCRs for LACK is higher than that for MPER.

## 4 Discussion

In general, co-delivery of an antigen with an immunostimulator in a particulate system is an effective way to generate a strong immune response, mediating efficient activation of innate immune cells and antigen presentation to APCs with a dose sparing effect [41], [42], [43], [44]. While physical association of B cell antigen peptides and liposome in vaccine formulation is required to augment antibody titers [45], [46], the effect of the manner in which CD4 T cell helper peptides are incorporated into liposome vaccines on the magnitude and the quality of the target antigen-specific cellular and antibody responses has not been well described. This is especially important considering that the helper peptides often induce their own peptide-specific antibody response. In this study, we used MPER/liposome vaccines as a model system to examine how the mode of the potent T helper peptide, LACK, formulation in liposome vaccine and its recognition by B cells can modulate antibody responses of the weakly immunogenic MPER antigen. We directly compared surface associated LACK to liposome via lipid anchored LACK, pLACK, with a formulation where soluble LACK peptide, sLACK, is present both encapsulated in the liposomes and freely in solution. The efficiency of sLACK incorporation into liposome is about 50% based on the amounts of sLACK remaining in solution after liposome incorporation measured by mass spectrometry analysis (data not shown). Given that 35% of sLACK is estimated to be attached to the preformed liposome surface in the post-extrusion method, approximately 15% of sLACK may be encapsulated into the aqueous interior, and free LACK peptides both attached to the surface of liposome and in the vaccine solution would likely dissociate from the MPER/liposome during migration to the regional LNs as previously observed with non-covalently associated MPER peptides to liposome [25]. Despite these incorporation differences, durable and high titer MPER-specific antibody responses were induced by both pLACK and sLACK formulated vaccines after two immunizations. Of note, a trend toward a reduced anti-MPER antibody titer induced by pLACK formulated vaccine was considerably more apparent at the lower LACK dose compared to that induced by the sLACK formulation. Consistent with these results, the frequency of MPER-specific GC B cells was higher than that of LACK-specific GC B cells in mice immunized with the sLACK formulated vaccine, whereas the opposite trend was observed in mice immunized with the pLACK formulated vaccine (Fig. 3). This differential indicates that when both MPER and LACK peptides are surface arrayed, LACK-specific B cells outcompete MPER-specific B cells for their survival and selection during GC reactions. It is evident that these GC survival differences mediate relatively disfavored induction of anti-MPER antibody responses, in particular at the lower dose of LACK (Fig. 5B). In addition, the increased MPER-specific antibody titers at the 40 μg dose of pLACK compared to that at the 10 μg dose of pLACK may be explained by increased T cell help by more LACK presentation on the MPER-specific B cells, compensating for the differences in B cell affinity as evidenced in others work that utilized anti-DEC-205 to deliver ovalbumin to specific B cell populations [30]. Therefore, the magnitude of the MPER-specific cellular and serological immune responses was modulated by the surface exposure of LACK as well as the quantity of LACK peptides. Our results are similar to previous observations seen with carrier protein induced suppression of polysaccharide antigen-specific antibody responses where a population of clonal B cells specific to the carrier exhibit suppression with an effective strength inversely correlated with dose [47].

While a slightly higher MPER-specific antibody titer was induced by sLACK formulated liposome vaccines compared to pLACK, a clear trend favoring relatively higher affinity serum antibodies specific to both MPER and LACK (Fig. 5C) was evident with surface associated pLACK immunizations. This may indicate the generation of relatively lower affinity antibodies induced by sLACK than that by pLACK formulated liposome vaccines. BCR affinity of GC B cells increases over time in a phenomenon known as affinity maturation, which is the major function of GC reaction. B cells harboring BCR with higher affinity acquire more antigen from follicular dendritic cells and present a larger amount of surface MHC II–peptide to Tfh cells, thereby outcompeting low-affinity B cells for obtaining more T cell help signal [14], [32]. The relatively greater affinity maturation of LACK-specific antibodies compared to that of MPER-specific antibodies (Figs. 5C and 6C) is likely to be contributed by the antigen affinity-based competition for T cell help. On the other hand, the increased antibody affinities for MPER- and LACK-specific B cells were positively correlated with the dose of LACK as well as pLACK formulation.

While the effect of preferential T cell help for the LACK-specific B cells cannot be excluded as the mechanism driving MPER-specific B cells affinity maturation, the induction of higher affinity antibodies by the pLACK compared to sLACK formulated vaccine may be in part explained by the amounts of pLACK peptides retained in direct association with MPER/liposome particles as an immune complex on the follicular dendritic cells in the GC. Covalent liposome association in the pLACK formulated vaccine improves relative loading capacity of helper peptides and the co-delivery of MPER and LACK peptides to the regional LN. Therefore, it is postulated that the enhanced co-delivery of pLACK and MPER in one liposome to the same B cells may result in presenting greater MHC II/LACK to Tfh cells compared to that by sLACK formulated liposome, which may cause dissociation of the LACK peptides from MPER containing liposome particles. This is consistent with MPER- and LACK-specific antibody affinities enhanced by higher dose of LACK (Fig. 5C). The results above also suggest that the effect of LACK formulation on the quantity and the quality of MPER-specific antibody responses may be greater when CD4 T cell antigen is limiting.

IgG subclasses produced in response to infection can dramatically affect the ability of humoral immunity to confer protection against disease. Studies showed that the properties of liposomes such as size, lipid composition and antigen encapsulation methods, etc. can skew Th1/Th2 antibody responses [28], [29], [48], [49], [16]. In our experimental setting, the only variable is the methods of LACK peptide formulation in liposome vaccine. Whereas, the MPER-specific antibody response was biased to the IgG1 subclass immune response in mice immunized with sLACK formulated liposome vaccine, balanced MPER-specific IgG1 and IgG2_a_ subclass antibody responses were induced by the pLACK formulated liposome vaccine. On the other hand, the LACK-specific IgG response skewed towards an IgG2_a_ subclass generated by the pLACK formulated vaccine (Fig. 5D), indicating that both the LACK formulation and the intrinsic nature of the antigen sequence itself may influence the IgG subclass distribution. Previously, studies suggested that cognate B cell antigen affinity and antigen dose played a role in regulating the level of isotype switching [50], [51], [52]. In that regard, helper peptide formulation in particulate vaccines may regulate antigen-specific IgG isotype responses by influencing the amounts of helper peptides available for T cell help in competition with target antigen-specific B cells.

In conclusion, our results show that the methods for CD4 T cell helper peptide incorporation in liposome vaccines influence the immunogenicity of B cell target antigen. While covalent attachment of helper peptides increases the loading capacity of the helper peptides in liposome vaccines, and supplying more antigen for T cell help, both the dose of antigen and the innate BCR affinity of naïve B cells for the helper peptide arrayed on the surface of liposome may play an important role in modulating the magnitude and the quality of target antigen specific B cell immune responses.

Neutralization requires serum IgG concentrations to be of sufficient affinity and abundance. In that regard, the quality of the elicited antibodies - such as antibody affinity, specificity and/or or neutralizing capacity - has been identified as a determining factor in efficacy. The lack of neutralizing activities in immune sera elicited by MPER/liposome vaccine requires further modifications of current MPER immunogens to correctly mimic quaternary structure of MPER on the virion surface. However, given sequence variations in diverse isolates of HIV-1 permitting virus escape from immune protection, the elicitation of broad and potent neutralizing antibodies by MPER/liposome vaccine will benefit from co-delivery of a well designed MPER immunogen physically associated with an optimized dose of helper peptides.
