# Supplementary material for: Co-delivery of a CD4 T cell helper epitope via covalent liposome attachment with a surface-arrayed B cell target antigen fosters higher affinity antibody responses
Source: Vaccine. 2018 Oct 1;36(41):6191–201. doi: 10.1016/j.vaccine.2018.08.014 (PMC6192012; doi:10.1016/j.vaccine.2018.08.014)
Supplement: Supplementary file 1 [file mmc2.pdf]

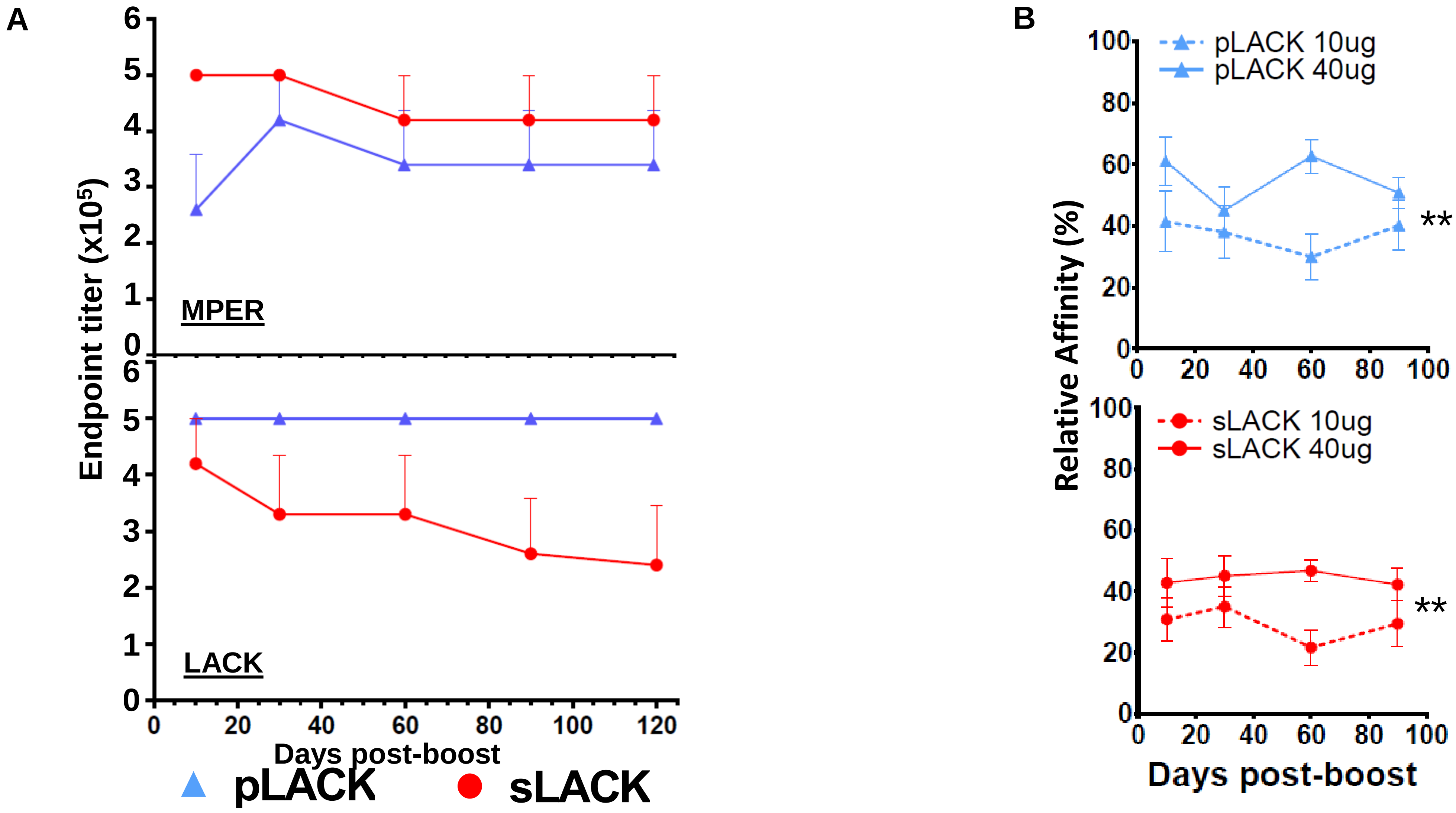

Supplemental Figure 1. Comparison of temporal and dose kinetics within sLACK and pLACK immunized mice. (**A**) Anti-MPER IgG (top) and anti-LACK (bottom) IgG ELISA titers quantified as the reciprocal of the dilution generating signal two times above background, elicited by 40µg sLACK-formulated (red circles) MPER/liposome vaccine compared with those elicited by 40µg pLACK-formulated (blue triangles) MPER/liposome vaccine at 10, 30, 60, 90, and 120 days post-boost. (Data is derived from curves shown in **Fig. 5A**) (**B**) Direct comparison of the effect of LACK dose (10µg, dashed line and 40µg, solid line) on MPER-specific IgG affinity maturation. Data is identical to that shown in (**Fig. 5C**); however, plotted data and statistics rearranged for direct comparison of dose rather than LACK formulation. Statistical analysis by two-way ANOVA with p-value for dose comparision shown (**$p<0.01$). Top graph shows pLACK (blue triangles) and bottom is sLACK (red circles) results. Absorbance values used for calculating the ratio were specifically determined by finding the absorbance at the high density MPER or LACK/liposome (peptide to lipid ratio of 1:50) EC50 and using that EC50 dilution to find the absorbance given on the low density MPER or LACK/liposome (1:1000) curve.
