# Supplementary material for: Co-delivery of a CD4 T cell helper epitope via covalent liposome attachment with a surface-arrayed B cell target antigen fosters higher affinity antibody responses
Source: Vaccine. 2018 Oct 1;36(41):6191–201. doi: 10.1016/j.vaccine.2018.08.014 (PMC6192012; doi:10.1016/j.vaccine.2018.08.014)
Supplement: Supplementary file 2 [file mmc1.pdf]

Table S1. 4E10 B cells staining exp.:

mAb	Clone	Catalog #	company
Fc block Anti-Mouse CD16/CD32	2.4G2 (RUO)	553142	BD Biosciences
Anti-Human Ig $_k$ mAb, PE	TB28-2	12-9970-42	eBioscience
LACK1:I-A^d biotin	2C44	---	Made in house
Streptavidin, Alexa Fluor™ 647 Conjugate, AF 647	---	S32357	Thermo Fisher Scientific
DAPI	---	D9542	Sigma-Aldrich

Table S2. GC B cells:

mAb	Clone	Catalog #	company
Fixable Viability Dye eFluor 506	---	65-0866-14	Thermo Fisher Scientific
MPER/liposome-biotin & LACK/liposome-FITC	---	Refer to M+M	Made in house
Fc block Anti-Mouse CD16/CD32	2.4G2	553142	BD Biosciences
CD19 (eBio1D3 (1D3)), PE-Cy7	eBio1D3 (1D3)	25-0193-82	eBioscience
CD38, APC	90	102711	Biolegend
GL7, PE	GL7	144608	Biolegend
Streptavidin, BV421	---	563259	BD Biosciences
Anti-mouse IgD, APC/Cy7	11-26c.2a	405715	Biolegend

Table S3. GC Tfh/Tfr cells:

mAb	Clone	Catalog #	company
Fixable Viability Dye eFluor 506	---	65-0866-14	Thermo Fisher Scientific
Fc block Anti-Mouse CD16/CD32	2.4G2 (RUO)	553142	BD Biosciences
CD185 (CXCR5) Biotin	SPRCL5	11-0193-82	eBioscience
CD279 (PD-1), PE	J43	12-9985-82	eBioscience
CD4, APC-eFluor 780	RM4-5	47004282	eBioscience
CD25, APC	PC61	102011	Biolegend
CD278 (ICOS), PE-Cy7	7E.17G9	25-9942-82	eBioscience
FOXP3, Alexa Fluor 488	FJK-16s	53-5773-82	eBioscience

Table S4. LACK presenting B and DCs:

mAb	Clone	Catalog #	company
Fc block CD16/CD32	2.4G2 (RUO)	553142	BD Biosciences
CD19, PE-Cyanine7	eBio1D3 (1D3)	25-0193-82	eBioscience
CD45R/B220, APC/Cy7	RA3-6B2	103223	Biolegend
CD11c, FITC	N418	117305	Biolegend
MHCII, A647	39-10-8	115009	Biolegend
CD86, PE	GL1	105007	Biolegend
LACK:I-A^d	2C44	---	Made in house
Streptavidin, BV421	---	563259	BD Biosciences
